# Bone phenotype of patients with genetic forms of lipodystrophy: a systematic review of literature

**DOI:** 10.1210/clinem/dgag256

**Published:** 2026-07-06

**Authors:** Elif Kusgozoglu, Daniela Q C M Barge-Schaapveld, Patrick C N Rensen, Tim Roek, Hester Vlaardingerbroek, Ingrid M Jazet, Natasha M Appelman-Dijkstra

**Affiliations:** Department of Internal Medicine, Division of Endocrinology, Leiden University Medical Center, 2333 ZA Leiden, The Netherlands; Department of Internal Medicine, Division of Endocrinology, Center for Rare Endocrine and Bone Conditions, Leiden University Medical Center, 2333 ZA Leiden, The Netherlands; Department of Clinical Genetics, Leiden University Medical Center, 2333 ZA Leiden, The Netherlands; Department of Internal Medicine, Division of Endocrinology, Leiden University Medical Center, 2333 ZA Leiden, The Netherlands; Einthoven Laboratory for Experimental Vascular Medicine, Leiden University Medical Center, 2333 ZA Leiden, The Netherlands; Department of Internal Medicine, Division of Endocrinology, Leiden University Medical Center, 2333 ZA Leiden, The Netherlands; Einthoven Laboratory for Experimental Vascular Medicine, Leiden University Medical Center, 2333 ZA Leiden, The Netherlands; Department of Clinical Genetics, Leiden University Medical Center, 2333 ZA Leiden, The Netherlands; Department of Pediatrics, Division of Pediatric Endocrinology, Willem Alexander Children's Hospital, Leiden University Medical Center, 2333 ZA Leiden, The Netherlands; Department of Internal Medicine, Division of Endocrinology, Leiden University Medical Center, 2333 ZA Leiden, The Netherlands; Department of Internal Medicine, Division of Endocrinology, Center for Rare Endocrine and Bone Conditions, Leiden University Medical Center, 2333 ZA Leiden, The Netherlands; Department of Internal Medicine, Division of Endocrinology, Leiden University Medical Center, 2333 ZA Leiden, The Netherlands; Department of Internal Medicine, Division of Endocrinology, Center for Rare Endocrine and Bone Conditions, Leiden University Medical Center, 2333 ZA Leiden, The Netherlands

**Keywords:** genetic lipodystrophy, familial lipodystrophy, CGL, FPLD, osteoporosis, bone metabolism

## Abstract

**Objective:**

Congenital lipodystrophies (LDs), including congenital generalized LD (CGL) and familial partial LD (FPLD), are rare disorders characterized by absent or abnormal adipose tissue distribution, leading to clinical, hormonal, and metabolic complications. Although these systemic features are well described, data on bone involvement are limited. This literature review summarizes current evidence on bone manifestations in genetic LDs and potential underlying mechanisms.

**Methods:**

A systematic search was performed in PubMed, EMBASE, and the Cochrane Library using terms related to LD and bone outcomes. No restrictions on date, language, or study design were applied. Studies reporting bone-related outcomes in genetic LD in humans were included. Study quality was assessed using PRISMA guidelines and the JBI checklist.

**Results:**

Of 2632 studies identified, 15 met the inclusion criteria. Most focused on CGL1 and CGL2; no studies were found on CGL3 or CGL4. Reported bone abnormalities in CGL1 and CGL2 included osteolytic lesions, bone cysts, osteosclerosis, and pseudo-osteopoikilosis, predominantly in long bones. Bone mineral density (BMD) was generally normal to high at trabecular sites. In contrast, FPLD2 was characterized by reduced BMD at cortical sites and lower trabecular bone score, suggesting impaired bone quality. Proposed mechanisms include reduced bone marrow adipose tissue, disrupted marrow fat conversion, and altered endocrine signaling.

**Conclusion:**

Bone abnormalities are characteristic yet often underrecognized features of genetic LDs, particularly CGL1 and CGL2. Current evidence underlines the broad skeletal impact of adipose dysfunction and highlights the need for standardized, genetically stratified studies to clarify bone risk and guide clinical management in congenital LD.

Lipodystrophy (LD) is a rare heterogeneous disorder that is characterized by a complete (generalized) or partial loss of subcutaneous adipose tissue, leading to ectopic fat storage as well as disruption in the release of adipose tissue-derived hormones, such as leptin, adiponectin, and resistin. Ultimately, this leads to a range of clinical, hormonal, and metabolic complications associated with insulin resistance, leading to glucose intolerance, diabetes mellitus (DM), hypertriglyceridemia, and metabolic dysfunction-associated steatotic liver disease, as well as effects on bone metabolism.

The underlying cause and clinical manifestations of LD vary by its subtype, which includes congenital and acquired forms (1-5). Genetic forms of LD include congenital generalized lipodystrophy (CGL) and familial partial lipodystrophy (FPLD), which both consist of multiple subtypes (6, 7). Of these, Dunnigan (FPLD2) is the most common type, based on a pathogenic variant in the *LMNA* gene. CGL, also known as Berardinelli-Seip syndrome, is a rare autosomal recessive disease with an estimated prevalence of 1.3-4.6 per million (2, 8), although true prevalence is uncertain due to underdiagnosis. It leads to a total or almost total loss of subcutaneous adipose tissue. It is typically diagnosed early in childhood and is classified into 4 subtypes according to gene variants responsible for the formation and development of adipocytes from birth: CGL1 (*AGPAT2* gene), CGL2 (*BSCL2* gene), CGL3 (*CAV1* gene), and CGL4 (*PTRF* gene). CGL1 and CGL2 are the most frequently reported subtypes of CGL in the literature (9, 10). FPLD is a rare autosomal dominant disease that typically manifests after puberty and is more frequently diagnosed in women due to sex-related differences in fat distribution and disease expression (6, 7). In FPLD2, loss of subcutaneous adipose tissue typically affects the limbs, buttocks, and hips, with relative or increased fat accumulation in the face, neck, and trunk. Thus, CGL and FPLD2 differ in genetic cause, age of onset, pattern of fat distribution, and overall disease severity (6).

Genetic LD constitutes a unique metabolic milieu characterized by near or complete absence or dysfunction of adipose tissue with plausible downstream effects on bone. Altered amount and/or function of bone marrow adipose tissue (BMAT) may influence bone mineral density (BMD), while disruption of adipokine signaling (notably low leptin) and insulin resistance/hyperinsulinemia may further contribute to altered bone metabolism. The literature reports reduced BMD, altered bone architecture, and increased fracture risk in some FPLD cohorts (11). Conversely, patients with CGL may display normal or even increased bone mass and osteosclerotic features, consistent with animal models demonstrating high bone mass in the absence of adipose tissue (12). Nevertheless, findings across studies remain heterogeneous and mechanistically incompletely understood. This systematic literature review aims to provide an overview of genetic LD (CGL and FPLD) and its impact on bone health and underlying mechanisms.

## Methods

We conducted a systematic literature search with the assistance of a trained librarian. The following databases were queried in November 2024 with an updated search in December 2024: PubMed, Embase, and the Cochrane Library. The search terms included keywords like “lipodystrophy,” “bone density,” “osteomalacia,” “bone marrow,” “bone remodeling,” “bone resorption,” “fracture,” “osteoporosis,” and “osteopenia”, along with Medical Subject Headings (MeSH) terms including “lipodystrophy,” “bone,” and “bones”. The complete search strategy is provided in Supplementary Appendix (13). Boolean operators (“AND”, “OR”) were used to refine the search term and ensure a comprehensive identification of relevant articles. The literature search was conducted without applying any filters, such as publication date, language, or study type. Two researchers (E.K. and N.M.A.-D.) independently conducted the search at different times and selected the articles based on title and abstract. Considering the rarity of the disorder, all articles addressing congenital forms of LD and their impact on bone health in humans were included. Studies were excluded if they did not report measurable outcomes related to bone health or were published in a language other than English. The review was conducted and reported in accordance with the PRISMA 2020 statement and JBI methodology for systematic reviews. The study selection process is shown in Supplementary Figure 1 (13). The PRISMA checklist and completed JBI critical appraisal checklists for all included studies are available in the Supplementary Appendix and Supplementary Files 1-15, respectively (13). Due to the limited number and heterogeneity of available studies, a meta-analysis was not feasible. All relevant studies were included.

## Results

The search term retrieved 2632 articles. After removing duplicates and screening titles, abstracts, and full texts, a total of 15 English-language articles concerning congenital LD and bone manifestations in adults were included. Most of the studies (*n* = 12) discussed bone manifestations in CGL1 and CGL2, while no studies addressed bone effects in CGL3 or CGL4. Two studies addressed FPLD (FPLD2, *n* = 1; FPLD1 and FPLD2, *n* = 1), and 13 studies addressed CGL1 and CGL2, 2 of which did not specify the CGL subtype. Most studies were cross-sectional (*n* = 5), followed by literature reviews (*n* = 3), case series (*n* = 3), case reports (*n* = 2), a longitudinal cohort study (*n* = 1), and a quasi-experimental study (*n* = 1). An overview of included studies, skeletal findings, and proposed pathophysiological mechanisms is provided in Supplementary Tables 1-4 (13).

### CGL1 and CGL2

Bone manifestations are common in CGL. Thirteen articles have reported skeletal effects in CGL1 and CGL2 patients. In 1992, Fleckenstein et al systematically described diffuse skeletal abnormalities in the appendicular skeleton, including abnormal bone marrow space and the absence of bone marrow fat in long bones. This absence was associated with a predisposition to focal osteolytic lesions and cyst formation during adolescence (14). An earlier study also reported cystic bone lesions in the epiphyses of long bones in 5 of 6 adults with CGL. Bone biopsies showed spongy osseous tissue with thin trabeculae, irregular contours, and increased cement lines, suggesting abnormal bone remodeling. The cysts consisted of capillary-rich tissue with thin walls and serous content, whereas cystic lesions were absent in infants (15).

A recent cross-sectional study of 19 patients aged 8-42 years found osteolytic lesions in 86% of CGL1 and 40% of CGL2 patients. These lesions were typically simple or multicystic, bilateral and symmetrical (86%), most commonly affecting the femur (53%), particularly the epiphysis and metaphysis, followed by hands and feet. Osteosclerosis was the second most frequent manifestation, present in 42% (*n* = 8) of CGL1 patients, involving both axial and appendicular skeleton, with homogeneous spinal sclerosis and cortical thickening of long bones. No osteosclerosis was observed in CGL2 patients, and none of these findings were associated with pain (16).

A retrospective observational case series study over 38 years, using ultrasonography and computed tomography (CT), reported skeletal changes in 7 patients, all showing advanced skeletal age (>3 years). Early childhood findings included sclerotic lesions, increased bone density, and enlarged epiphyses; later, trabecular coarsening, osteolytic lesions, and cysts appeared. One patient developed a pathological ulnar fracture at age 23. Across the included studies, only isolated cases of fractures associated with osteolytic lesions were reported, without consistent evidence of instability or increased fracture susceptibility across studies. Bone cysts were reported predominantly in CGL1, especially in long bones, and correlated with hepatopathy (β = 0.76) and DM onset (β = 0.47), suggesting systemic disease involvement (17). This finding is consistent with the reported timing of metabolic abnormalities after adolescence.

Case reports also describe increased lumbar spine and femoral neck BMD in severe CGL cases (18). In a retrospective study of 24 LD patients, 3 patterns were identified in CGL (*n* = 10): diffuse osteosclerosis (70%), osteolytic lesions (80%), and pseudo-osteopoikilosis (∼40%, only in CGL1). Lesions were mostly bilateral and symmetrical, affecting the proximal femur, tibia, humerus, metacarpals, and iliac bones. Osteosclerosis involved both trabecular and cortical bone, with cortical thickening and narrowing of medullary cavities. One CGL1 patient developed symptomatic osteolytic lesions complicated by fracture and osteoarthritis. Longitudinal data are scarce, but progressive lumbar sclerosis and later osteolytic lesions have been reported in individual cases (19, 20).

Regarding BMD, Lima et al reported that 12 of 21 patients with CGL1 and CGL2 had normal or elevated BMD (Z-score > + 2.5) at one or more sites, with higher Z-scores reported at sites such as the lumbar spine and ultradistal radius. Lower Z-scores were observed at the radius (33% region; −0.5 SD), indicating site-specific variation in BMD (10). All sites had mean Z-scores above zero, except the radius 33% (−0.5 SD). The ultradistal radius showed the highest Z-score (+3.4 SD). Elevated BMD was more prominent in CGL2 and at trabecular sites (10). In contrast, Freire et al reported 79% of CGL1 patients and 40% of CGL2 patients had an increased Z-score of greater than +2.5, and this appeared higher in CGL1 patients compared to CGL2 patients, mainly in the lumbar spine and total body (16). No data were reported on skeletal phenotypes in CGL3 or CGL4.

In FPLD, radiographic abnormalities consisted of predominantly nonspecific degenerative changes (eg, osteoarthritis, calcific tendonitis, and osteochondrosis), without the osteolytic or osteosclerotic bone lesions typically described in CGL (19).

### FPLD1 and FPLD2

Two cross-sectional studies have investigated bone manifestations in FPLD. Pombo et al observed that FPLD2 patients had lower fat content in the lower limbs, trunk and total body compared to FPLD1 and nonlipodystrophic control patients as measured by DXA scans. In FPLD1 patients, fat in lower extremities was lower than the nonlipodystrophic control group. Regarding BMD, there was no significant difference in total or segmental BMD between FPLD1, FPLD2, and nonlipodystrophic control groups after adjusting for age, height, fat mass, and fat-free mass (FFM). Of the FPLD2 patients, 70.6% had pathogenic variants in exon 8 in *LMNA*. Additionally, no significant differences in BMD were observed between FPLD patients carrying LMNA pathogenic variants in exon 8 and those with variants in other exons. KöB index was used to stratify FPLD1 patients according to the severity of lower-limb fat loss; however, BMD did not differ between patients with low (<3.477) vs high KöB index (>3.477). Postmenopausal women with LD had lower unadjusted BMD compared to premenopausal women, even after adjusting for height, fat, and FFM (21).

Similar findings were observed in a study by Moreira et al, reporting no significant differences in BMD or Z-scores (lumbar spine, femoral neck, and total hip) between FPLD2 patients and controls, except in the one-third radius, where FPLD2 patients had lower BMD. In addition to BMD, bone microarchitecture was assessed in one study using trabecular bone score (TBS), which was significantly lower in FPLD2 patients compared to controls (*P* < 0.01), suggesting impaired bone quality (22). No additional studies using advanced imaging techniques such as HR-pQCT were identified within the included literature. Additionally, osteoclast differentiation was increased in the FPLD2 group after stimulation of peripheral blood mononuclear cells with RANKL, resulting in an increased osteoclast area, suggesting increased bone resorption. After adjustment for DM, sex, age and BMI, osteocalcin showed a negative association with lumbar spine BMD (β = −1.04, *P* < 0.05). Furthermore, the study reported significantly higher BMAT in FPLD2 patients compared with matched controls (22).

## Discussion

Genetic forms of LD are rare, and studies assessing their bone manifestations are limited. This systematic literature review summarizes the available evidence on skeletal abnormalities in genetic forms of LD. Bone involvement has been reported most consistently in CGL and is characterized by structural bone changes such as osteosclerosis, osteolytic lesions, and pseudo-osteopoikilosis, often affecting the long bones and sometimes associated with cystic lesions. Regarding BMD, a paradoxical pattern emerges, with increased or normal BMD in CGL1 and CGL2 and normal to reduced BMD in partial LD, such as FPLD2. These findings underscore the complex relationship between adipose tissue loss, metabolic dysfunction, and bone remodeling. Importantly, the included studies are highly heterogeneous in terms of design, patient populations, imaging modalities, and skeletal sites assessed. As a result, this review should be interpreted as primarily descriptive in nature. Quantitative comparisons between studies and causal inferences regarding underlying mechanisms are not possible based on the currently available evidence.

### Bone manifestations

Across the reviewed literature, bone abnormalities were described in a relatively consistent manner, despite small sample sizes and methodological heterogeneity. The presence of osteosclerotic and osteolytic lesions suggests that bone remodeling in CGL is profoundly altered. In most cases, the lesions were localized to the epiphyses and metaphyses of long bones and, over time, were frequently associated with cyst formation. Although longitudinal data are scarce, some reports suggest that sclerotic bone changes may predominate in childhood and evolve into osteolytic or mixed lesions in adulthood. This transition may reflect altered bone remodeling driven by metabolic and structural changes in the bone marrow environment.

The absence of BMAT may represent one of the important contributors to this pathophysiology. In healthy individuals, BMAT plays a regulatory role in bone homeostasis by providing paracrine support for osteoblasts and secreting cytokines that influence the balance between bone formation and bone resorption. In CGL, the congenital lack of marrow fat (more in CGL2 compared to CGL1) may disrupt this balance. Normally, red marrow is progressively replaced by yellow (fat-rich) marrow during childhood (23). Imaging studies in CGL suggest impaired marrow fat development and persistence of hematopoietic tissue, findings that have been proposed to contribute to osteolytic changes and cyst formation (14, 15). Importantly, while BMAT is markedly reduced or absent in CGL1 and CGL2, it does not appear to be systematically absent in FPLD2. Although direct measurements are limited, available evidence from a limited number of studies suggests that BMAT is not absent and may even be increased in FPLD2. Consequently, the proposed shift toward osteoblast differentiation in generalized LD may be less pronounced in FPLD2. In addition, metabolic disturbances such as insulin resistance and altered adipokine profiles, which are generally more severe in CGL, may negatively affect bone quality. These differences in BMAT preservation and metabolic disturbances may contribute to the different skeletal phenotypes observed between generalized and partial LD and may help explain why BMD is often normal or reduced in FPLD2, in contrast to the increased BMD frequently observed in CGL.

In addition to the lack of BMAT, several mechanisms could hypothetically contribute to bone abnormalities in CGL. First, the absence of local adipocytes may deprive osteoblasts of paracrine growth signals that promote balanced bone formation. Second, increased mechanical loading from muscular hypertrophy, a common feature of CGL1 and CGL2, may stimulate periosteal bone formation and contribute to the sclerotic phenotype. Third, abnormal vascularization and persistent hematopoietic activity could alter bone metabolism and mineral distribution. Together, these factors may create an environment that promotes both abnormal bone deposition and focal resorption, explaining the coexistence of osteosclerosis and osteolytic lesions in CGL patients. Importantly, while alterations in BMAT are supported by imaging and histopathological evidence, several other proposed mechanisms remain speculative and are primarily based on indirect evidence. Therefore, their relative contribution to the observed skeletal phenotype should be interpreted with caution. Only a limited number of studies have directly assessed or specifically characterized BMAT, primarily through imaging techniques such as magnetic resonance imaging (MRI) (eg, Fleckenstein et al). Most available evidence regarding BMAT in LD is therefore indirect and based on inferred alterations in marrow composition rather than direct quantification.

### Bone mineral density

CGL1 and CGL2 show a BMD pattern that is opposite to that observed in FPLD2, whereas FPLD2 patients may show reduced cortical BMD and altered trabecular microarchitecture. In CGL1 and CGL2, BMD is often reported as normal or increased, with elevated values observed at sites such as the lumbar spine, a predominantly trabecular region (10, 16, 18, 24, 25). In several of the included studies, specific *LMNA* mutations were reported, including p.Arg482Trp, p.Arg482Gln, and other variants such as p.Asn466Asp, p.Ile299Val, p.Arg644Cys, and p.Ser583Leu. However, mutation reporting was not uniform across all studies, and considerable genetic heterogeneity was observed. Moreover, none of the studies systematically assessed genotype-phenotype correlations with skeletal outcomes, limiting conclusions on the relationship between specific *LMNA* mutations and skeletal manifestations. *LMNA* mutations are also known to cause a spectrum of disorders, including progeroid syndromes, which are characterized by premature aging and skeletal abnormalities (26-28). Although progeroid features were not systematically reported in the included studies, this overlap may be relevant, as accelerated aging processes could contribute to early alterations in bone quality. However, data specifically addressing this relationship in LD remain limited.

Several metabolic mechanisms may explain these findings. First, the absence of BMAT may enhance bone mass. BMAT is increasingly recognized as a regulator of bone formation (25, 29-31). Bone marrow adipocytes and osteoblasts share a common progenitor: the mesenchymal stem cell. In the absence of adipogenic differentiation, these precursor cells are more likely to commit to the osteoblastic lineage, promoting bone formation and potentially increasing BMD. Furthermore, adipocytes in the marrow secrete pro-inflammatory cytokines, such as TNF-α and IL-6, which stimulate osteoclast activity and bone resorption (32, 33). Their absence may therefore suppress bone resorption and favor net bone formation.

Second, metabolic factors such as hyperinsulinemia and hypoleptinemia appear to play important roles. Hyperinsulinemia, commonly observed in CGL1 and CGL2 patients, has anabolic effects on bone through stimulation of osteoblast proliferation and differentiation via the activation of the MAPK pathway and upregulation of osteogenic gene expression (9). Insulin also promotes bone formation by increasing collagen synthesis and enhancing osteocalcin production. In contrast, congenital LDs without insulin resistance have been associated with osteopenia, emphasizing the bone-protective effect of insulin (34). Notably, none of the studies reported osteocalcin levels, since osteocalcin and insulin influence each other in bone formation. High osteocalcin levels have been associated with lower insulin resistance (35).

Hypoleptinemia, another feature of CGL, may further contribute to increased bone mass (36). Experimental animal studies have demonstrated that reduced leptin signaling is associated with increased bone mass (37). Leptin acts both centrally and peripherally on bone metabolism. Centrally, leptin inhibits bone formation via hypothalamic pathways involving β_2_-adrenergic signaling in osteoblasts (38), whereas peripherally it may stimulate osteoblast differentiation and bone formation through direct effects on osteoblast and osteoclast activity (39). In CGL, leptin deficiency may suppress the central inhibitory pathway more strongly than the peripheral anabolic effects, potentially leading to a net increase in bone formation and higher BMD. The balance between these central and peripheral effects may help explain the observed high bone mass despite leptin deficiency. In addition, adiponectin, another adipokine altered in LD, may also contribute to bone metabolism. Adiponectin has been shown to influence both osteoblast and osteoclast activity and has been associated with increased bone resorption and lower BMD in several clinical studies. In LD, altered adiponectin levels may therefore further contribute to bone remodeling. However, its precise role in the skeletal phenotype of LD remains incompletely understood. Nonetheless, leptin's effects are modulated by sex, hormonal state, and BMI, which are variables that were rarely reported in the reviewed studies, most of which predominantly included female participants (40).

Additional contributors may include muscle hypertrophy and sex hormone alterations. Increased lean mass in CGL enhances mechanical loading on bones, which stimulates bone formation through mechanotransduction (10). Hyperinsulinemia may also reduce sex hormone-binding globulin (SHBG) levels, leading to increased bioavailable sex steroids that further promote bone formation (41). However, this mechanism may not fully explain the elevated BMD observed even in children with CGL, suggesting that other metabolic and developmental factors are involved.

Interestingly, differences between CGL1 and CGL2 subtypes may account for some of the variability in BMD findings. Genotype-phenotype heterogeneity between AGPAT2- and BSCL2-related disease has previously been described, including differences in metabolic severity, age of onset, and clinical manifestations (42-44). In CGL1 and especially CGL2, adipose tissue deficiency is more severe and may involve the bone marrow compartment, whereas in CGL3 and CGL4, marrow fat distribution appears to be more preserved. This may explain why increased BMD is predominantly observed in CGL1 and CGL2. Studies comparing subtypes have reported conflicting results, with some suggesting higher BMD in CGL2 and others in CGL1, likely reflecting small sample sizes and unbalanced subtype distribution (10, 16). These findings should therefore be interpreted as preliminary trends rather than definitive subtype-specific differences, particularly given the small sample sizes, heterogeneous study designs, and unbalanced subtype distribution across studies. Although CGL3 and CGL4 are recognized subtypes of CGL, no included studies specifically reported skeletal manifestations or bone phenotypes in these subtypes. From a molecular perspective, these subtype differences may be explained by the specific genetic defects underlying each form. *AGPAT2*, the gene mutated in CGL1, is required for proper adipose differentiation; however, *AGPAT2* reduction does not abolish adipogenesis uniformly across all fat depots (2, 45). Consequently, BMAT can still be partially retained in some CGL1 patients, possibly due to compensatory activity of other *AGPAT* isoforms that are more related to nonmetabolically active adipose depots. In contrast, *BSCL2* deficiency, causing CGL2, disrupts adipogenesis more profoundly and globally, including within the bone marrow compartment. Thus, while *AGPAT2* is essential for adipocyte formation, its loss does not entirely preclude adipogenesis in all tissues, consistent with the presence of residual BMAT (3, 45). However, this hypothesis remains largely inferential, as direct BMAT quantification was not performed in most studies. Given the key regulatory role of BMAT in bone metabolism, differences in marrow fat preservation between CGL1 and CGL2 could represent a more plausible explanation for the observed variability in BMD, together with the proposed mechanical and hormonal factors. Future studies should therefore include direct BMAT assessment (eg, by MRI or MR spectroscopy) to clarify its contribution to skeletal differences between different CGL types. Moreover, trabecular sites such as the lumbar spine show greater BMD increases than cortical sites like the distal radius, indicating site-specific differences in bone response.

Importantly, these findings further support the concept that BMD alone may not adequately reflect bone quality or strength. Reports of reduced TBS and altered microarchitecture in FPLD2 suggest that bone quality may be impaired despite preserved or even elevated bone mass. This apparent dissociation between BMD and bone quality highlights the need for more detailed imaging approaches, such as high-resolution peripheral quantitative CT, to evaluate true bone integrity in lipodystrophic syndromes. No cases of bone malignancy were reported in the studies included in this review.

### Clinical implications

Despite the consistent radiological characterization of skeletal abnormalities in genetic LDs, their clinical significance remains incompletely understood. Across the available literature, osteolytic lesions, cystic changes, and osteosclerosis are frequently described, particularly in patients with CGL (16, 19). However, most of these abnormalities appear to be largely asymptomatic and are often incidentally detected on imaging (19).

Importantly, data on clinically relevant outcomes such as bone pain, functional impairment, and fracture risk are scarce. Only isolated cases of fractures associated with osteolytic lesions have been reported, without consistent evidence of increased fracture risk across studies (19). Overall, the relationship between imaging findings and bone fragility remains unclear.

Furthermore, the observation of normal or increased BMD in several studies, particularly in CGL, complicates the interpretation of skeletal risk, as higher BMD does not necessarily reflect preserved bone quality or strength. This concept is supported by emerging data on bone microarchitecture in FPLD, where reduced trabecular bone score has been reported despite largely preserved BMD, suggesting that alterations in bone quality may occur independently of bone mass.

From a clinical perspective, no standardized treatment strategies for skeletal manifestations in LD have been established. Management is therefore primarily focused on optimizing metabolic control, including treatment of insulin resistance and, where applicable, leptin replacement therapy. Given the largely asymptomatic nature of most skeletal lesions, routine intervention is generally not required. However, clinical monitoring and orthopedic evaluation may be needed in selected cases with structural complications such as fractures.

### Future perspectives

The available evidence remains limited by small and overlapping cohorts, heterogeneous diagnostic criteria, and the absence of genetic confirmation in several studies. Moreover, biochemical markers of bone turnover were not systematically assessed across studies. As a result, insights into bone formation and bone resorption dynamics remain limited, further restricting interpretation of the underlying mechanisms of skeletal abnormalities. Few studies applied standardized imaging protocols or assessed bone metabolism markers. As a result, the natural history of skeletal abnormalities in LD, including how metabolic derangements interact with bone development and remodeling, remains insufficiently understood.

Future research should focus on larger, genetically well-defined cohorts, and include systematic longitudinal assessments of bone structure, bone density, and bone metabolism. Standardized imaging (DXA, MRI, HR-pQCT) combined with biochemical markers of bone turnover would allow for better characterization of the bone phenotype and improved understanding of disease progression from childhood to adulthood. Prospective studies such as the ECLip cohort may provide critical insights into causal pathways linking metabolic dysfunction, adipose tissue loss, and skeletal changes, as well as identify early predictors of bone complications (46). In addition, research should extend beyond the frequently studied subtypes (CGL1/2 and FPLD1/2) to include the full genetic spectrum of congenital and familial partial LDs. This will be crucial for understanding subtype-specific skeletal manifestations and their underlying molecular mechanisms, including potential roles for disrupted adipokine signaling, ectopic lipid accumulation, and altered osteoblast-osteoclast dynamics.

Finally, the clinical implications of these bone abnormalities should be investigated. Clarifying their impact on fracture risk, bone pain, physical functioning, and quality of life will inform screening and management strategies. Understanding the interaction between fat, bone, and metabolism in LD may also enhance our understanding of more common metabolic diseases, in which similar pathways may be dysregulated.

## Conclusion

Bone involvement in genetic LDs represents a distinct and complex skeletal phenotype driven by the interplay between adipose tissue deficiency, metabolic dysfunction, and alterations in the marrow environment. The near absence of BMAT may represent one of the key mechanisms linking fat loss to both structural abnormalities and, paradoxically, elevated BMD, while hyperinsulinemia, hypoleptinemia, and mechanical factors may further modulate bone remodeling across CGL (primarily CGL1 and CGL2) and FPLD subtypes. Importantly, increased BMD does not necessarily translate into preserved bone quality or reduced fracture risk. Clinically, improved recognition of these skeletal manifestations could enhance diagnostic precision, support future risk stratification, and facilitate earlier intervention to prevent functional impairment. However, current evidence is limited by small, heterogeneous cohorts and inconsistent phenotyping. Addressing these gaps will require genetically stratified, longitudinal studies that integrate advanced imaging, biochemical markers of bone turnover, and detailed metabolic profiling. Such efforts are essential to define the true burden and mechanisms of bone disease and identify potential therapeutic targets. Insights gained from these rare disorders may also clarify shared pathways underlying skeletal fragility in more common metabolic conditions.

## Data Availability

Data sharing is not applicable to this article as no new datasets were generated or analyzed during this study.

## Abbreviations

BMAT: bone marrow adipose tissue
BMD: bone mineral density
CGL: congenital generalized lipodystrophy
CT: computed tomography
DM: diabetes mellitus
FFM: fat-free mass
FPLD: familial partial lipodystrophy
LD: lipodystrophy
MRI: magnetic resonance imaging
SHBG: sex hormone-binding globulin
TBS: trabecular bone score
